# Srs2 and Pif1 as Model Systems for Understanding Sf1a and Sf1b Helicase Structure and Function

**DOI:** 10.3390/genes12091319

**Published:** 2021-08-26

**Authors:** Aviv Meir, Eric C. Greene

**Affiliations:** Department of Biochemistry & Molecular Biophysics, Columbia University, New York, NY 10032, USA; am5377@cumc.columbia.edu

**Keywords:** Sf1a helicase, Sf1b helicase, Srs2, Pif1, DNA repair, genome integrity

## Abstract

Helicases are enzymes that convert the chemical energy stored in ATP into mechanical work, allowing them to move along and manipulate nucleic acids. The helicase superfamily 1 (Sf1) is one of the largest subgroups of helicases and they are required for a range of cellular activities across all domains of life. Sf1 helicases can be further subdivided into two classes called the Sf1a and Sf1b helicases, which move in opposite directions on nucleic acids. The results of this movement can range from the separation of strands within duplex nucleic acids to the physical remodeling or removal of nucleoprotein complexes. Here, we describe the characteristics of the Sf1a helicase Srs2 and the Sf1b helicase Pif1, both from the model organism *Saccharomyces cerevisiae*, focusing on the roles that they play in homologous recombination, a DNA repair pathway that is necessary for maintaining genome integrity.

## 1. Introduction

Helicases are a class of nucleic acid motor proteins that use the energy derived from ATP hydrolysis to translocate along DNA or RNA substrates [1,2,3,4]. This ability to move along nucleic acids allows helicases to fulfill a diverse range of cellular functions involving nucleic acid metabolism, including the unwinding of duplex or structured nucleic acids and the remodeling or disruption of other nucleoprotein complexes [1,2,3,4,5,6,7,8,9,10]. There are nearly 100 identified helicases encoded within the human genome, and these proteins participate in almost all aspects of nucleic acid metabolism [1,2,3,4,5,6,7,8,9,10]. Importantly, mutations in helicase genes involved in DNA repair processes have been linked to numerous human diseases in which genomic instability, immunodeficiency, mental retardation, premature aging, and predisposition to cancer are common features [7,11,12,13,14,15,16,17].

**Figure 1:**
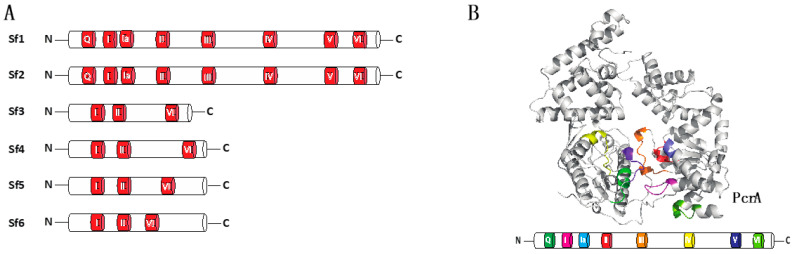
Conservation of motifs in helicases super families. (**A**) Diagrams illustrating classification of helicase super families and depiction of conserved motifs in each superfamily. (**B**) Crystal structure of PcrA helicase (PDB:1QHH) and diagram emphasizing the distribution of the conserved motifs along the helicase structure.

All helicases contain a core domain comprised of two RecA–like folds, which couple ATP binding and hydrolysis to protein conformational changes that mobilize the helicase along nucleic acids [2,3,10,18]. Helicases can be divided into six superfamilies, based on a set of conserved helicase motifs within the core domain (Figure 1A) [3,19,20]. Superfamily I (Sf1) is one of the largest and most diverse group of helicases and Sf1 helicases can be subdivided into two groups based on the direction of translocation: Sf1a helicases move in the 3′→5′ direction relative to the bound strand of nucleic acid and Sf1b helicases move in the opposite direction [1,2,3,4].

Here, we discuss the structure, function, and regulation Sf1 helicases using *Saccharomyces cerevisiae* Srs2 and Pif1 as representatives of prototypical Sf1a and Sf1b helicases. *S. cerevisiae* Srs2 and Pif1, and closely related homologs, have been studied extensively at the genetic and molecular level, thus offering insight into the similarities and differences between the Sf1a and Sf1b helicases. We compare their functions and molecular mechanisms, focusing on their roles in homologous DNA recombination. We also highlight future queries that will be necessary to better understand the mechanisms and functions of these crucial motor proteins.

## 2. Helicase Molecular Mechanisms

### 2.1. Helicase Domains and Motifs

Helicases can be divided into six super families, termed Sf1 through Sf6 (Figure 1A) [3,19,20,21]. A characteristic feature of helicases is the presence of highly conserved amino acid sequence motifs (Figure 1B), and the amino acid sequence identity of their conserved helicase motifs defines each of the six super families [22,23,24,25]. These motifs are clustered within a core region that is approximately 200 to 700 amino acid residues in length, and they are separated from one another by regions of low sequence conservation [26]. The Sf1 helicase core domain contains at least seven conserved amino acid motifs (termed motifs Q, I, Ia, II, III, IV, V and VI; Figure 1B) [3,22,27,28,29]. A key feature of the helicase core domain is its ability to bind and hydrolyze ATP and couple these ATP hydrolysis cycles to movement along nucleic acids [1,2,3,4]. In contrast to the highly conserved core domain, the N-terminal domain (NTD) and the C-terminal domain (CTD) flanking the helicase core exhibit a high degree of sequence and length variability. The divergent NTDs and CTDs are responsible for individual protein functions, whereas the highly conserved motifs are involved in ATP binding and hydrolysis and the binding and unwinding of nucleic acid substrates. Sf1 and Sf2 helicases are typically monomeric, whereas the Sf3 through Sf6 helicases often form oligomers (typically hexamers) that contain a central channel through which DNA can pass [3,10]. Although monomeric, some helicases in both the Sf1 and Sf2 superfamilies can act in concert on the same strand of nucleic acid [30,31]. One potential advantage of these so called “helicase trains” is that the collective action of multiple helicases may confer enhanced translocation characteristics, such as greater processivity or an increased capacity to disrupt stable nucleic acid structures or nucleic acid-bound proteins.

### 2.2. General Aspects of Helicase Translocation

**Figure 2:**
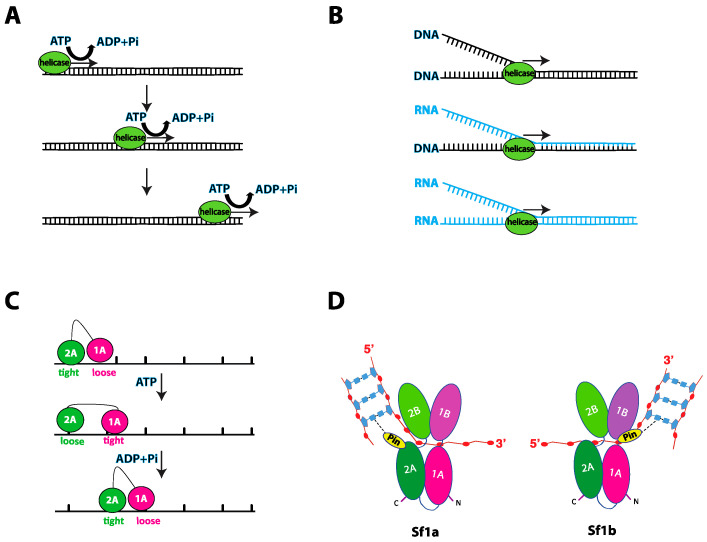
Multiple faces of helicases activity. (**A**) Schematic of helicase motion showing movement in a specific direction on nucleic acids coupled to ATP hydrolysis. (**B**) Schematics of nucleic acid duplex unwinding, specifically DNA-DNA, DNA-RNA, and RNA-RNA, by helicases. DNA strands are colored black while RNA stands are colored light blue. (**C**) Illustration of the inchworm mechanism. Tandem RecA-like domains (magenta and green circles) make up the motor domain of the helicase. The motor domain interacts with the ssDNA track through DNA-binding domains, which alternate between tightly and loosely bound states. ATP binding is coupled to rotation of the RecA-like domains and changes in the ssDNA affinity of the DNA-binding domains, thus moving the helicase in a specific direction. (**D**) Illustration of the four subdomains in Sf1a and Sf1b, highlighting the presence of the wedge/pin domain located in the 2A subdomain for Sf1a and 1A subdomain for Sf1b. The wedge/pin domain interacts with the nucleic acid at the ds-ssDNA junction, aiding in the unwinding of the dsDNA.

Helicases function by converting the chemical energy stored in the ATP molecule into mechanical work, resulting in unidirectional movement along a nucleic acid strand (Figure 2A). This translocation activity can result in the separation of strands within duplex nucleic acids or the disruption of nucleoprotein complexes (Figure 2B). A crucial issue within the field is understanding exactly how helicases move on nucleic acid substrates. The most common mechanism proposed for helicase translocation is known as the “inchworm” mechanism, initially derived from the structural studies of the two bacterial Sf1a helicases UvrD and PcrA, both of which are closely related to *S. cerevisiae* Srs2 [32,33,34,35]. The inchworm mechanism involves two points of contact between the helicase and the nucleic acid. These two points of contact undergo cycles of alternating nucleic acid binding affinity between “loose” and “tight” bound states (Figure 2C). Binding affinities are tightly coupled to the ATP hydrolysis cycle, enabling the protein to move along the nucleic acid in one nucleotide increments (Figure 2C) [32]. It is likely that these general mechanistic principles for converting the chemical energy of ATP into motion along a nucleic acid are shared among many helicases.

### 2.3. Nucleic Acid Unwinding

Intense effort has been focused on understanding precisely how helicases unwind their nucleic acid substrates. Numerous studies have revealed the existence of helicase structural domains that act as mechanical elements that can help drive strand separation (see below). These structures are often called “separation pins” or “wedges” and can range from simple β-hairpins, such as is found in UvrD [32,33], to more complex domains such as in the case of the heterotrimeric RecBCD complex [36,37]. Indeed, separation pins or wedges have been identified in many helicases, including Sf1 helicases such as the heterotrimeric RecBCD complex from *Escherichia coli*, Deinococcus radiodurans RecD2, Bacillus stearothermophilus PcrA, *E. coli* Rep and *E. coli* UvrD as well as in the SF2 helicases *Thermatoga maritima* RecG, *E. coli* RecQ, *Klebsiella pneumoniae* PriA and the hepatitis C viral RNA helicase NS3 [33,36,38,39,40,41,42,43,44]. Nucleic acid unwinding is achieved when the helicase core exerts a force on one strand of a duplex nucleic acid, thus pulling the duplex across the pin/wedge domain which in turn leads to mechanical separation of the two nucleic strands (Figure 2D) [37].

## 3. Structural Features of Sf1a and Sf1b Helicases

In this section, we describe structural studies that have led to detailed understanding of the Sf1a and Sf1b helicases, with emphasis on seminal studies of PcrA, UvrD, and RecD2.

### 3.1. Structural Organization of Sf1a and Sf1b Helicases

**Figure 3:**
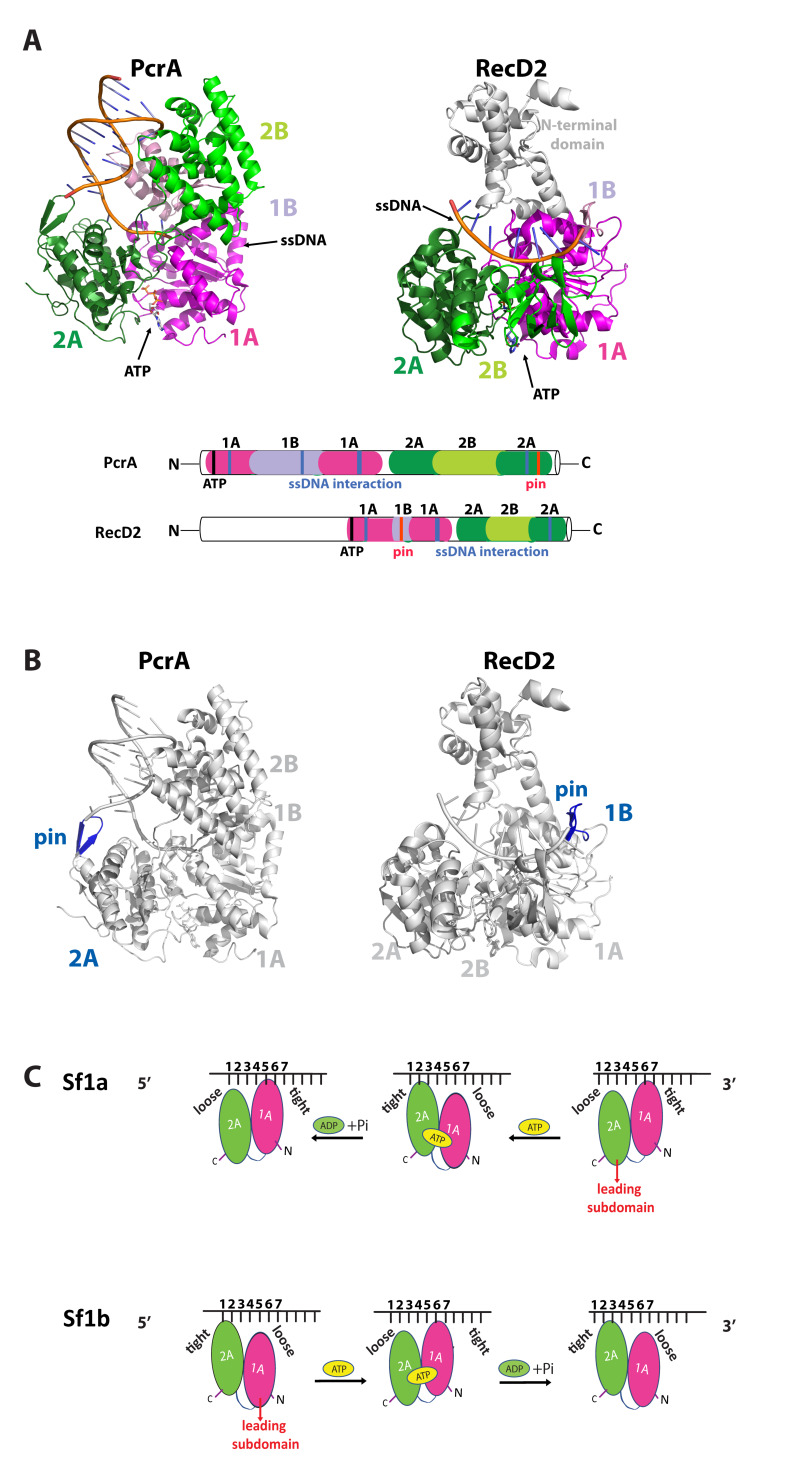
Structure and translocation mechanism of Sf1a and Sf1b helicases. (**A**) Crystal structures (left panel) and diagram (right panel) of PcrA (PDB:3PJR) and RecD2 (PDB:3GPL), highlighting tandem RecA domains (domain 1A—magenta, domain 1B—pink, domain 2A—dark green, domain 2B—light green), ATP binding pocket, and ssDNA binding cleft. (**B**) Crystal structures of PcrA (PDB:3PJR) and RecD2 (PDB:3GPL) in grey, highlighting the pin domain in blue located at 2A subdomain of PcrA and 1B subdomain of RecD2. (**C**) Models for Sf1a 3′ to 5′ movement (top panel) and Sf1b 5′ to 3′ movement (bottom panel). Both Sf1a and Sf1b employ a characteristic inchworm mechanism, leading with subdomains 2A and 1A, respectively. Numbers signify individual DNA bases.

The Sf1 helicase core is comprised of four globular domains (1A, 2A, 1B and 2B), which together resemble a pair of tandem RecA-like folds with a single ATP-binding pocket residing in the center between domains 1A and 2A (Figure 3A) [35,38,45]. The conserved helicase motifs are clustered together within these tandem RecA-like domains, forming the bipartite ATP-binding pocket and a large portion of the nucleic acid-binding cleft (Figure 3A). Interestingly, the pin domain that is responsible for strand separation is located on opposite sides of the helicase core for Sf1a and Sf1b helicases, consistent with the fact these enzymes travel in opposite direction along their nucleic acid substrates (Figure 3B) [46]. For PcrA of the Sf1a family, the separation pin is located within domain 2A and is positioned at the ssDNA/dsDNA junction well-poised to assist with strand separation [35]. For RecD2, a Sf1b helicase, the separation pin is located on the opposite side of the helicase core within domain 1B [47]. Importantly, a RecD2 mutant lacking the pin hydrolyzed ATP at the same rate as the wild-type protein, indicating that the motor activity of the helicase core domain was likely functional, but lacked the ability to unwinding dsDNA, thus uncoupling motor activity from DNA unwinding [47]. Similarly, mutation of the UvrD pin amino acid tyrosine 621 to alanine causes an ~50% reduction in its ability to unwind dsDNA [33].

### 3.2. Mechanism of Sf1a and Sf1b Helicase Translocation

Even though they share significant structural similarity, the Sf1a and Sf1b helicases translocate in opposite directions on their nucleic acid substrates (Figure 3C). Structural studies of the Sf1a helicases PcrA and UvrD and the Sf1b helicase RecD2 bound to DNA in the presence of various nucleotide cofactors have proven crucial for defining the atomic-level structural rearrangements that take place during helicase translocation [32,33,34,35,39]. Comparison of these structures shows that all three helicases bind to ssDNA in the same orientation with 2A domain oriented in the 5′ direction relative to the bound ssDNA and the 1A domain oriented towards 3′ side of the ssDNA (Figure 3C) [32,33,34,35,39]. The ssDNA-binding cleft runs across the top surfaces of the 1A and 2A domains [32,33,34,35,39]. Opening and closing of the cleft between the 1A and 2A domains in the presence and absence of ATP appears to provide conformational changes that are the means of translocation via an inchworm mechanism (Figure 3C) [32,33,34,35,39]. For PcrA bound to DNA in the absence of nucleotide, the cleft between domains 1A and 2A is open and domain 1A is tightly bound to the ssDNA [34,35]. ATP binding leads to closure of the cleft between domains 1A and 2A, while concurrently domain 1A releases its tight grip on DNA and domain 2A establishes tighter contact with the DNA [34,35]. Thus, cleft closure causes the DNA to slide in the 3′→5′ direction across the surface of domain 1A [34,35]. ATP hydrolysis leads to cleft opening while domain 1A re-establishes a tight grip on the DNA, whereas domain 2A weakens hold on DNA allowing the DNA to slide in the 3′→5′ direction across its surface [34,35].

In the case of RecD2, in the absence of ATP, the cleft between domains 1A and 2A remains open and domain 1A is more loosely bound than domain 2A (Figure 3C). ATP binding causes cleft closure, while at the same time domain 2A relaxes its grip allowing it to slide in the 5′→3′ towards domain 1A, which maintains tighter contact with the DNA [39]. ATP hydrolysis causes the cleft between the 1A and 2A domains to re-open, domain 1A now relaxes its grip on the DNA to slide across its surface in the 5′→3′ direction [39]. Thus, similar to PcrA and UvrD, protein conformational changes (opening and closing) and changes in the relative DNA-binding affinity of two contact surfaces are coupled to the ATP binding and hydrolysis cycle, allowing RecD2 to move along the DNA via an inchworm mechanism, albeit with the opposite polarity [35]. In simple terms, for the Sf1a helicases, domain 1A always chases domain 2A along the DNA, whereas for the Sf1b domain 2A always chases domain 1A along the DNA (Figure 3C). In all cases, the structural data are most parsimonious with a translocation step size of a single nucleotide [33,35,39]. Note that for the sake of simplicity, we have described the relative motions and changes in DNA contacts of just domains 1A and 2A, and for additional details we refer readers to the original studies [33,35,39].

## 4. Srs2 and Pif1 as Model Systems for Understanding Sf1a and Sf1b Helicases

In the sections below we highlight several genetic, biochemical, and biophysical studies that have been used to obtain insights into the biological roles and mechanisms of *S. cerevisiae* Srs2 and Pif1. For Srs2, we focus on its roles in homologous recombination; and for Pif1, we focus on its roles in BIR and replication termination. For further information on other activities of these helicases, we refer readers to several excellent reviews [1,6,48,49,50,51,52].

### 4.1. Srs2 Is an Sf1a Helicase That Regulates Homologous Recombination

The SRS2 gene was originally identified in a genetic screen for mutants that suppressed the sensitivity of rad6 mutants to DNA-damaging agents [53] and was later shown to harbor canonical helicase motifs [54]. Insights into Srs2 function were revealed by the finding that *srs2* mutants often exhibited a hyper-recombination phenotype, suggesting that Srs2 may play a role in restraining homologous recombination [55,56,57,58]. The *SRS2* gene encodes an Sf1a helicase that is closely related to the bacterial helicases Rep, PcrA, and UvrD [59]. Srs2 appears to be both structural and functional homolog of bacterial UvrD as both proteins seem to fulfill similar biological roles in genome maintenance (see below), thus the structural and mechanistic studies of *E. coli* UvrD have direct bearing upon our understanding of Srs2 [60,61]. In addition, *srs2* null alleles exhibit either synthetic lethality or slow growth phenotypes in combination with many genes involved in genome maintenance, including *RAD50*, *MRE11*, *SGS1*, *RAD54*, *RRM3*, *XRS2*, *CTF4*, *CTF8*, *MRC1*, *TOF1*, *MMS4*, *MUS81*, *RAD27*, *POL32* [62,63,64,65,66,67]. In many instances, deletion of the *RAD51* gene, which encodes a key recombinase necessary for homologous recombination (HR), alleviates the synthetic lethal or slow growth phenotypes, further implicating Srs2 as a central regulator of HR [54,59,62,63]. Biochemical studies confirmed that Srs2 (1174 amino acids; 134 kDa) has ssDNA-dependent ATP hydrolysis activity (*k*_cat_ ∼300 s^−1^) and can unwind duplex DNA [68,69]. Indeed, a single point mutation in the Srs2 Walker A ATP-binding motif (e.g., K41A or K41R) was sufficient to inactivate both its ATP hydrolysis and helicase activities [70].

### 4.2. Srs2 as Prototypical “Antirecombinase”

**Figure 4:**
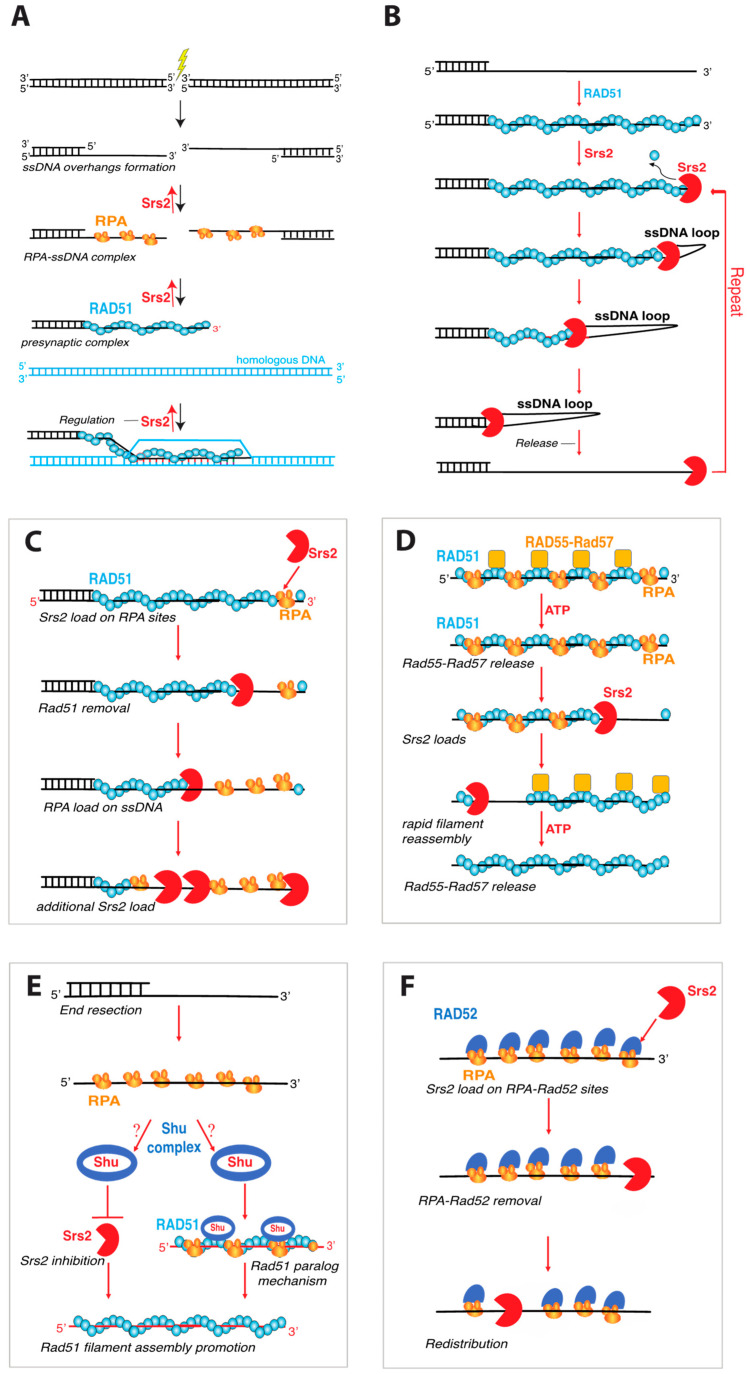
Srs2 antirecombinase activities during Homologous Recombination. (**A**) Schematics of Homologous Recombination (HR). Here, we present an overview of the HR mechanism and indicate the steps at which Srs2 has been implicated to act upon recombination intermediates. HR begins with the formation of a double-stranded break (DSB). (1) The break is first resected to form long 3′ ssDNA overhangs. (2) These ssDNA overhangs serve as a binding platform for replication protein A (RPA), which is necessary to protect the ssDNA against degradation and is also required for the removal of secondary structure to enable efficient assembly of the Rad51 filaments. (3) RPA is replaced by the Rad51 to yield Rad51-ssDNA filaments, also known as presynaptic complexes. The presynaptic complex then performs homology search to locate a double-stranded DNA molecule with sequence complementarity to the ssDNA that is bound by Rad51. (4) Finally, Rad51 catalyzes a strand invasion reaction to generate a D-loop intermediate, in which the presynaptic ssDNA is paired with its complement and the noncomplementary is displaced. As highlighted in the figure, Srs2 has been reported to act at three key stages of this pathway, by either dismantling RPA or Rad51 filaments, or by disrupting early strand invasion intermediates. (**B**) Repetitive shuttling of Srs2. Srs2 translocates on ssDNA along a ssDNA overhang, stripping off bound Rad51. Srs2 can repeatedly strip Rad51 from the ssDNA by either maintaining contact with the overhang by creating a small ssDNA loop or remaining at the junction and repeatedly catching and releasing the ssDNA. (**C**) Rad51 stripping from ssDNA initiates with Srs2 loading on RPA clusters. Rad51 is then removed from the ssDNA, allowing more RPA to bind and thus facilitating more Srs2 binding events. (**D**) Rad55-Rad57 stimulates rapid Rad51 filament assembly through transient binding interactions and then dissociates when Rad55 hydrolyzes ATP. The resulting Rad51 filaments are disrupted by Srs2. New Rad51 filaments are then re-assembled behind Srs2 through the stimulatory action of Rad55-Rad57. (**E**) The Shu complex might promote Rad51 filament formation by either inhibiting Srs2 recruitment to the break sites and preventing Srs2 inhibition of Rad51 filament formation or directly promoting Rad51 filament formation in a manner similar to Rad55-Rad57. (**F**) Srs2 Strips Rad52-bound RPA from ssDNA (stripping of Rad52 prebound RPA is not shown).

One of the most well characterized roles of Srs2 is its ability to regulate HR by removing the Rad51 recombinase from ssDNA recombination intermediates (Figure 4A) [1,6,71,72]. This type of “antirecombinase” activity has now also been demonstrated for several other helicases, including *E. coli* UvrD [61], *S. cerevisiae* Sgs1 [73], and the human helicases FBH1, PARI, BLM, RECQ5 [74,75,76,77,78,79,80]. As indicated above, several genetic studies revealed that Srs2 played a crucial role in constraining HR, and the mechanisms by which Srs2 accomplished this task was revealed in two biochemical studies which demonstrated that purified recombinant Srs2 could physically strip Rad51 from ssDNA [71,72]. Srs2 does not appear to dismantle paired D-loop intermediates, but instead acts earlier on the Rad51–bound ssDNA, which in turn prevents D-loop formation and subsequent DNA strand exchange [71,72]. Indeed, electron microscopic analysis showed Rad51 was physically removed from the ssDNA by Srs2 [71,72]. Subsequent studies showed that the Walker A box mutants Srs2–K41A and Srs2–K41R exhibited a loss of ATP hydrolysis and helicase activities and were unable to displace Rad51 from ssDNA [70]. In addition, yeast strains harboring these *srs2* mutations were highly sensitive to the DNA damaging agent, methyl methane sulfonate (MMS) and exhibited synthetic lethality in combination with either *sgs1* or *rad54* deletions [56,62,81]. These findings provided a clear link between the ATP-dependent motor activity of Srs2 and its role in removing Rad51 from DNA.

These studies raised the important question of what molecular principles might underly the ability of Srs2 to remove Rad51 from the ssDNA. In considering this question, it is important to understand the relationship between DNA-binding affinity and ATP binding and hydrolysis for the Rad51/RecA family of proteins. *S. cerevisiae* Rad51 is a member of the Rad51/RecA family, all of which are ATP-dependent DNA-binding proteins that form long right-handed helical filaments on ssDNA and promote the DNA transactions that are a central aspect of HR [82]. Importantly, the ssDNA–binding affinity of Rad51 and other Rad51/RecA family members is directly related to their ATP binding and hydrolysis cycle [82,83,84,85,86,87]. These proteins have a high affinity for DNA in their ATP–bound state, but a much lower affinity for DNA when in their ADP–bound state. Thus, ATP hydrolysis can lead to Rad51 filament disassembly. An ensemble biochemical study using fluorescence-based assays revealed that Srs2 takes advantage of the Rad51 ATP hydrolysis cycle to promote Rad51 dissociation from ssDNA [88]. This study proposed a model in which Srs2 bound to ssDNA would translocate in the 3′→5′ direction [88]. Upon making physical contact with the 3′ terminal monomer of a Rad51 filament, Srs2 would enhance the ATP hydrolysis activity of Rad51, thus promoting its release from DNA and allowing Srs2 to proceed to the next Rad51 monomer (Figure 4A) [88]. A key implication of this model is that Srs2 may allosterically stimulate Rad51 ATP hydrolysis activity. The Rad51 Walker A box mutant K191R, which is proficient for ATP binding but not ATP hydrolysis, can bind to DNA tightly but has a drastically reduced rate of dissociation, consistent with its inability to hydrolyze ATP [88,89,90]. As predicted from the model, Srs2 can remove Rad51–K191R from ssDNA but at a ~3–fold reduced rate compared to wild-type Rad51 [31,88].

### 4.3. Single-Molecule Studies of Srs2 Antirecombinase Activity

Given its importance to genome integrity and its function in Rad51 filament disruption, Srs2 has gained the attention of single molecule biophysicists with an interest in motor protein functions. Single-molecule fluorescence resonance energy transfer (smFRET) studies have shown Srs2 can undergo repetitive shuttling on short substrates that have an ssDNA/dsDNA junction (Figure 4B), which is similar to the shuttling behavior reported for numerous helicases [91]. The proposed mechanism for repetitive shuttling is that there is a DNA-binding site on Srs2 that can remain in contact with the 3′ ssDNA end allowing the motor domain to undergo translocation independently of the 3′ end-binding [91,92]. This results in the formation of a small ssDNA loop and enables the enzyme to maintain constant contact with the ssDNA substrate even upon dissociation of the motor domain (Figure 4B). An interesting implication of this repetitive shuttling behavior is that it may enable Srs2 to repeatedly clear Rad51 from ssDNA without dissociating into solution (Figure 4B) [91].

Studies using DNA curtains to visualize GFP–tagged Srs2 as it interacts with Rad51–bound ssDNA filaments have also proven useful and provided direct measures of Srs2 velocity and processivity [31,93]. These experiments revealed that Srs2 can translocate in the 3′→5′ direction at a velocity of ∼140 nucleotides per second (nt/s), corresponding to the removal of ~50 Rad51 monomers per second, over an average distance 20 kilo-nucleotides (knt) [31]. Interestingly, Srs2 did not load within the Rad51 filaments themselves, but instead loaded at short patches of RPA near the ends of the Rad51 filaments (Figure 4C) [31]. Srs2 translocation led to Rad51 removal, and the resulting naked ssDNA was quickly occupied by more RPA, which in turn enabled more efficient Srs2 loading behind the “pioneer” helicase (Figure 4C). Srs2 translocated more rapidly on both naked ssDNA (∼460 nt/s) and RPA-bound ssDNA (~180 nt/s) compared to Rad51-bound ssDNA (~140 nt/s), allowing for the accumulation of multiple Srs2 molecules behind the lead helicase, consistent with a “train” of Srs2 molecules acting upon the Rad51-ssDNA (Figure 4C) [31,93].

### 4.4. Regulation of Srs2 Antirecombinase Activity

The Rad51 paralog complex Rad55-Rad57 has emerged as an important regulator of Srs2. Rad51 paralogs are proteins that share ~20% identity with the conserved central ATPase core domain of Rad51 but have little to no similarity outside of this region and do not form filaments or catalyze strand exchange [94,95]. *S. cerevisiae* Rad55-Rad57 is a stable heterodimer and deletion of either *RAD55* or *RAD57* sensitizes cells to ionizing radiation (IR), but this phenotype can be suppressed by Rad51 overexpression or the deletion of *SRS2*, suggesting that Rad55-Rad57 might play a role in Rad51 filament stabilization [96,97]. Interestingly, Rad51–I345T was isolated as a suppressor mutation that alleviates the sensitivity of *rad55* and *rad57* deletion strains to ionizing radiation [98]. Rad51–I345T assembles into filaments more rapidly than wild-type Rad51 but Rad51–I345T filaments can still be disrupted by Srs2, suggesting that the role of Rad55–Rad57 may be to enhance Rad51 assembly kinetics [31]. Early biochemical studies suggested that Rad55–Rad57 might form a stable co-component of the Rad51-ssDNA filament and act by physically blocking Srs2 translocation [99]. However, recent single-molecule studies have suggested that Rad55-Rad57 does not form a stable co-component of the Rad51 filament, but instead acts transiently to promote more rapid Rad51 filament assembly and then rapidly dissociates from the mature filaments [100]. Thus, this study supported a model in which Rad55-Rad57 counteracts Srs2 by promoting rapid Rad51 filament reassembly after the passage of Srs2 (Figure 4D) [100]. The Shu complex, comprised of Shu1, Shu2, Psy3, and Csm2, has also been implicated as a regulator of Srs2 [101]. Although mechanistic details remain to be elucidated, Shu1 and Psy3 are both RAD51 paralogues, so it is possible that the Shu complex may act similarly to Rad55-Rad57 (Figure 4E).

Several studies have also suggested that the recombination mediator protein Rad52 can act as a negative regulator of Srs2 [102,103,104]. Early studies demonstrated that Rad51 forms foci at sites of DNA repair and Rad51 foci formation is dependent upon the presence of Rad52 [105]. Interestingly, in the absence of Srs2, Rad51 foci can form even without Rad52, but the corresponding Rad51 foci are not recombination proficient [102]. Increased formation of Rad51 foci in a *srs2Δ rad52Δ* double mutant, relative to *rad52Δ* alone, suggests a reduced requirement for Rad52 when Srs2 was absent [102]. This study also showed that Rad52 protected Rad51 filaments from Srs2 disruption in in vitro strand exchange assays, further suggesting a regulatory interplay between Rad52 and Srs2 [102]. Later work showed that Rad52 stabilizes Rad51 filaments rendering them toxic when Srs2 is absent [104]. However, disruption of the Rad51-Rad52 interaction alleviates this toxicity, and Rad51 is still recruited to HO-induced DSBs and the breaks are repaired [104]. Moreover, this work showed that Rad52 interacts with Srs2 in biochemical pulldown assays, suggesting the existence of a direct protein-protein interaction [104]. However, Rad52 does not inhibit Srs2 ATP hydrolysis activity in vitro, arguing against a model in which Rad52 completely blocks Srs2 activity [104]. Thus, the authors of this study suggest that Rad52 prevents Srs2 from dismantling Rad51 filaments during the early stages of recombination; but upon completion of repair, Rad52 sumoylation weakens its interactions with Rad51, allowing Srs2 to act upon any remaining Rad51 filaments [104]. Interestingly, single-molecule studies have shown that Srs2 can readily remove both RPA and Rad52 from ssDNA in the absence of Rad51, indicating that Rad52 in and of itself may not act as a physical blockage to Srs2 (Figure 4F) [93]. So, if Rad52 acts by physically blocking Srs2 translocation, then one might infer that it must do so only after the arrival of Rad51.

### 4.5. Pif1 Is an Sf1b Helicase with Multifaceted Roles in DNA Replication

The *PIF1* gene was originally identified in *S. cerevisiae* in a screen for mutations that change the recombination frequency of tandemly arrayed repeats within mitochondrial DNA, and was therefore named after the resulting petite integration frequency phenotype (PIF1) [106]. *PIF1* was later independently isolated in a screen to identify genes that affect telomere length, providing an indication that the helicase had a nuclear function in addition to its role in mitochondria [107]. Indeed, analysis of the *PIF1* ORF reveals two in frame AUG codons separated by 40 codons: translation from the first AUG generates a protein with a mitochondrial localization signal, while proteins translated from the second AUG lack this signal and localize to the nucleus [108].

The *PIF1* gene encodes an Sf1b DNA helicase and Pif1 homologs have been identified throughout biology ranging from bacteria to humans [49,109,110,111]. Interestingly, while most organisms only encode one PIF1 family helicase, *S. cerevisiae* expresses two: Pif1 (97 kDa; 859 aa) and Rrm3 (81 kDa; 723 aa), which share 40% sequence identity within their helicase core domains. Pif1 also shares strong sequence homology to the bacterial protein RecD [112]. The homology between Pif1 and RecD includes not only the seven helicase motifs, but also three additional unique motifs of unknown functions, named motifs A, B and C, which cluster between the helicase motifs IV and V [112]. Pif1 helicases also contain a 21 amino acid Pif1 signature sequence located between helicase motifs II and III [50,113]. This sequence is composed of an α-helix and a turn and is located at the entrance to the DNA binding site, opposite from the strand separation pin, and helps maintains a key phenylalanine residue (F71) in the appropriate position to assist with DNA (or DNA-RNA hybrid) strand separation [114,115]. In vitro experiments have confirmed that Pif1 exhibits ATP-dependent helicase activity and exhibits 5′→3′ translocase activity that enables it to unwind duplex DNA structures [116], G quadraplexes [117,118,119], and RNA-DNA hybrids [119,120,121]. Interestingly, Pif1 unwinds RNA-DNA hybrids better than duplex DNA, suggesting the possibility that it may participate in R-loop processing [121].

### 4.6. Pif1 Has Multifaceted Roles in DNA Replication

**Figure 5:**
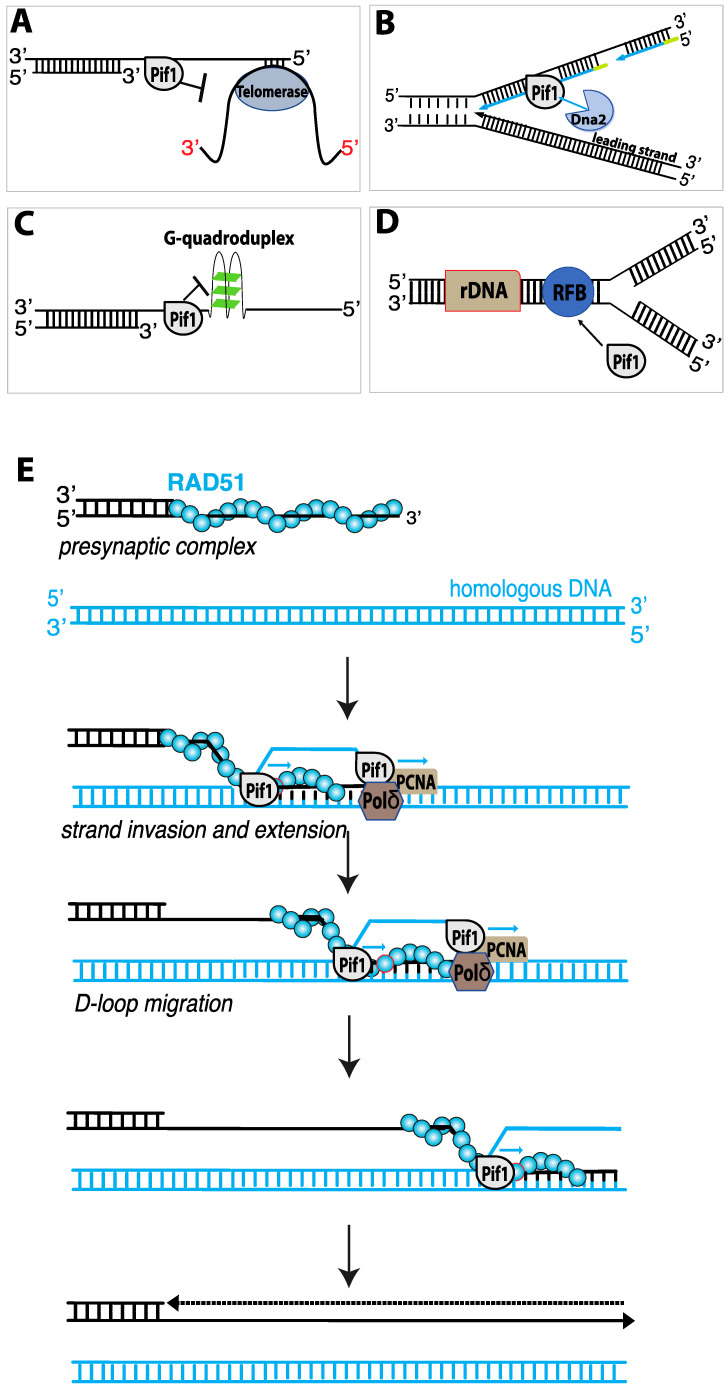
Multifaceted roles for Pif1 in nucleic acid metabolism. (**A**) Pif1 inhibits telomerase-mediated telomere elongation by directly removing telomerase from a DNA end [108]. (**B**) Pif1 contributes to DNA replication by affecting Okazaki fragment maturation by generating long flaps that are cleaved by the nuclease activity of Dna2 [123]. (**C**) Pif1 functions to maintain genome stability by blocking the formation of G- quadruplex structures [117]. (**D**) Pif1 inhibits fork progression at the replication fork barrier within ribosomal DNA (rDNA), ensuring that replication and transcription happen in the same direction through rDNA repeats. (**E**) Schematics of Pif1 involvement in Break-induced replication. Break-induced replication (BIR) is a specialized homologous-recombination pathway for DNA double-strand break (DSB) repair induced by Pif1.

Pif1 participates in numerous aspects of DNA replication, including: telomere length regulation (Figure 5A) [107,122]; Okazaki fragment maturation (Figure 5B) [123,124]; assisting fork progression through difficult to replicate sites (Figure 5C) [117,125,126,127]; the maintenance of the replication fork barrier (RFB) within ribosomal DNA (Figure 5D) [128]; replication fork convergence during the completion of DNA synthesis [129]; and DNA synthesis during break-induced replication (Figure 5E) [130,131]. Below, we discuss these aspects of Pif1 biological function in more detail.

### 4.7. Pif1 and Telomere Length Regulation

The first indication that Pif1 played a role in telomere regulation were the findings that yeast cells lacking nuclear Pif1 or cells expressing *pif1* mutants had longer telomeres than wild-type cells, in contrast, Pif1 over-expression resulted in telomere shortening [94,122]. In vitro studies revealed that recombinant Pif1 could reduce telomerase processivity by displacing telomerase from DNA ends, where as an ATPase deficient Pif1 mutant (Pif1-K264A) had no effect [132]. One of the most distinct telomere phenotypes of Pif1 deficient cells is the destiny of DSBs. In wild-type cells, DSB are most commonly repaired by HR and only rarely repaired by de novo telomere addition. However, in *pif1Δ* or *pif1-m2* cells (which have mitochondrial Pif1 but no Pif1 nuclear localization), the rate of de novo telomere addition can increase by up to almost 1000-fold [107,133,134]. Thus, a normal function of Pif1 is to downregulate de novo telomere addition to newly generated DSBs [134].

### 4.8. Pif1 and Replication Fork Convergence

The processes of replication initiation and elongation have been studies for many years using reconstituted reaction systems comprised of purified *S. cerevisiae* replication proteins. However, what takes place during the later stages of replication, when two forks must converge with one another, has remained largely unknown. Pif1 has very recently been identified as a factor that promotes the convergence of eukaryotic replication forks [129]. Using plasmid-based biochemical assays with *S. cerevisiae* replication proteins, it was shown that two converging replication forks stall to produce replication intermediates in which the nascent strands are approximately 90- to 190-bp shorter than the full-length substrate [129]. Similar stalling was observed even on linearized substrates, indicating that changes in DNA topology did not likely cause the problem. The researchers reasoned that a helicase absent in the reconstituted replication assays might be necessary to help synthesis of full-length products during these final stages of replication. Therefore, they tested yeast DNA helicases, including Pif1, Rrm3, Sgs1, Srs2, Dna2, and Chl1 to determine whether they allowed for synthesis of full-length replication products. Surprisingly, both Pif1 and its paralog Rrm3 could support the efficient synthesis of full-length replication products in vitro. Similarly, plasmids from *rrm3Δ pif1-m2* cells also yielded under-replicated intermediates due to defects in fork convergence during replication termination, and this defect was alleviated in cells deficient for just one of the helicases [129]. These exciting new findings reveal a new role for Pif1 (and Rrm3) in the late stages of replication termination.

### 4.9. Pif1 Acts as a “Pseudo-Replicative” DNA Helicase during BIR

One crucial function of Pif1 is its role in break–induced replication (BIR). Cells can use BIR for the repair of one-ended DSBs that arise at eroded telomeres or when a replication fork encounters a single-strand nick (Figure 5E) [135,136]. Although BIR can be used to repair one-ended DSBs, it is also highly mutagenic (~1000-fold higher than normal replication), and can lead to a loss of heterozygosity, chromosomal translocations, and copy number variations, all of which are hallmarks of cancer [136]. During BIR, Rad51 and Rad54 catalyze strand invasion to pair the broken DSB end with a homologous dsDNA template [136]. Notably, Pif1, which is not required for normal S-phase DNA replication, is essential for BIR [130,131,136,137]. Polymerase delta (polδ) drives DNA synthesis during BIR, and Pif1 is thought to help unwind the DNA in front polδ and also unwind the newly synthesized DNA strand from the template, thus allowing BIR DNA synthesis to occur within the context of a migrating DNA bubble-like structure (Figure 5E) [130,131]. Cells lacking Pif1 are deficient for BIR. While the initial steps of BIR can occur normally, polδ recruitment and DNA synthesis are substantially reduced and strand synthesis stalls within ~5 kb of the site of strand invasion [137]. Further evidence for the role of Pif1 in BIR comes from biochemical assays which have demonstrated that purified Pif1 can stimulate polδ-mediated DNA synthesis in vitro from D-loops made with Rad51, RPA and Rad54 [130]. In the absence of Pif1, polδ (in reactions with the DNA sliding clamp protein PCNA and the clamp loader complex RFC) could extend the D-loops by ~200–500 nucleotides, whereas inclusion of Pif1 allowed for the synthesis of thousands of nucleotides [130].

Several basics aspects DNA synthesis during BIR appears to be significantly different from the DNA replication that takes place during S-phase. For example, the rate of DNA synthesis during BIR (~0.5 kb/min) is approximately six-times slower than normal S-phase replication, although the exact reason for this difference remains unclear [137]. In addition, lagging strand synthesis in vivo is significantly delayed during BIR, resulting in the formation of tracts of ssDNA up to ~20–30 kilo-nucleotides in length [137]. Biochemical analysis of in vitro BIR replication products by restriction endonuclease digest, electron microscopy, and ChIP analysis of RPA binding also confirmed the production of an extensive amount of ssDNA, further suggesting that leading and lagging strand synthesis are decoupled during BIR [130]. The resulting ssDNA is highly susceptible to chemical mutagens, such as methyl methane sulfonate (MMS), perhaps explaining in part why BIR is itself highly mutagenic [131]. In addition, the ssDNA is also subject to promiscuous Rad51-driven strand invasion events that can lead to lethal recombination intermediates [55]. This latter problem is found in *srs2Δ* strains, thus revealing a crucial role for Srs2 in protecting BIR intermediates from unregulated recombination [55].

Finally, recent work has shown that Pif1 is also necessary for BIR in human cell lines [138]. Importantly, this study demonstrated that the breast cancer-associated Pif1 mutant L319P, which resides within the Pif1 family signature motif, was defective for BIR [138]. This work provides crucial evidence suggesting that not only is the role of Pif1 in BIR is likely to be broadly conserved among eukaryotes, but also providing a direct indication that BIR (and Pif1) may play roles in human genome integrity and cancers.

### 4.10. Single-Molecule Studies of Pif1 Activity

Pif1 has been the subject of several single molecule investigations. SmFRET studies of *S. cerevisiae* Pif1 have revealed that Pif1 monomers can bind to a 3′ ss/dsDNA junction very tightly (K_d_ ~ 7 nM) with no appreciable dissociation taking place over a 30-min time period [119]. These data also yielded characteristic sawtooth patterns in the FRET trajectories indicative that Pif1 translocation along a 3′ ssDNA tail can be coupled to repetitive DNA looping activity (referred to as “periodic patrolling”), very similar to the shuttling behavior reported for Srs2 and several other DNA helicases [91,92,119]. Analysis of the data were consistent with a translocation velocity of 85 nt/s with a 1 nucleotide step size [119]. Pif1 monomers could also unwind a 31-bp RNA-DNA hybrid, although the unwinding rate was surprisingly slow and required multiple attempts, taking 6.3 min at 20 µM ATP (1.6 min at saturating ATP), corresponding to 200 cycles of repeated attempts at unwinding the substrate [119]. Interestingly, Pif1 monomers were unable to unwind dsDNA duplex, but Pif1 “trains” could [119]. Consistent with bulk biochemical data, smFRET studies have also shown that Pif1 can unwind G quadraplexes and does so in a series of steps to yield a fully unwound DNA strand (Figure 5C) [119].

SmFRET studies have also been performed with forked DNA substrates [139]. Interestingly, these experiments revealed two classes of unwinding events: repetitive unwinding attempts and full substrate winding [139]. Sometimes single enzymes exhibited only repetitive unwinding; sometimes they transitioned to full unwinding; and sometimes they just completely unwound the substrate without the repetitive phase. These distinct modes of unwinding suggest the possibility that unidentified conformational transitions within Pif1 may regulate how it unwinds nucleic acids [139]. Finally, a study of *Thermus oshimai* Pif1 used smFRET to investigate how protein dynamics were coupled to enzyme translocation [140]. In this case, the donor and acceptor dyes were not located on the DNA substrate but were instead linked to the Pif1 itself. It was revealed that rotational motion between domain 1A and domain 2B, reflecting a structural transition found in the apo and DNA-bound ToPif1 crystal structures, was also coupled to enzyme translocation on DNA [140].

Two studies have also looked at Pif1 activities using magnetic tweezer-based assays [141,142]. The first of the two studies examined repetitive unwinding of hairpin structures at forces ranging from 4 to 7.5 pN and revealed that more force applied to the DNA assisted unwinding by Pif1, yielding both faster unwinding (from ~50 nt/s at low force up to 150 nt/s at higher force; measured at 100 µM ATP with a 40 bp hairpin) and greater processivity for individual unwinding events (from ~25 bp up to ~200 bp; measured at 500 µM ATP with a 270 bp hairpin) [141]. A second magnetic tweezer study yielded a Pif1 translocation velocity of 140 nt/s at 100 µM on DNA extended by a force of 17 pN and the authors estimated a maximum velocity of ~220 nt/s at saturating ATP [142]. Notably, this study reported that the majority of cases consisted of regular, unidirectional translocation events (~90%) whereas a smaller fraction of the population (~10%) exhibited repetitive translocation events [142].

As alluded to above, Pif1 plays a number of roles in DNA replication. An early effort to study the impact of Pif1 on DNA replication at the single molecule level used dCas9 as a barrier to polymerase δ (pol δ) [143]. This study found that the DNA replication was blocked by dCas9, allowing bypass in only 14% of cases [143]. In striking contrast, when Pif1 was present 78% of the replication forks were able to bypass dCas9 and there was no evidence of a pause when the fork encountered dCas9 [143]. Surprisingly, Pif1 supported fork bypass of dCas9 even in the absence of an interaction with PCNA, suggesting that within this in vitro setting Pif1 may have engaged the fork through its affinity for the DNA itself or perhaps some other protein [143]. Finally, this study revealed that Pif1 did not simply evict dCas9 from the DNA, instead dCas9 was evicted in only 30% of cases, while the remaining events led to dCas9 transfer to the either the leading strand (36%) or the lagging strand (33%).

## 5. Future Directions

Despite the great extent of our current knowledge, there remains much to be learned from studying helicases. From a mechanistic perspective, we still do not have a full appreciation of the protein conformational changes that couple ATP hydrolysis to protein motion. Nor do we understand the protein structural features that dictate the speed and processivity of a given helicase. We also have a limited understanding of the parameters that dictate the difference in directionality for the Sf1a and Sf1b helicases. For example, given our current understanding that directionality is dictated by coupling changes in protein structure during the ATP hydrolysis cycle with alternating grip of the protein at two sites on the DNA, would it be possible to reengineer a helicase to move in the opposite direction? What allows for the repetitive shuttling behavior reported in single molecule studies of so many helicases? The most reasonable explanation for these observations is the existence of a secondary DNA-binding site that would allow the helicases to remain in contact with a fixed point on the DNA while the helicase core translocates away from the location. The identification of mutants that specifically affect this hypothetical DNA-binding site could be most informative.

Understanding the mechanism of helicase regulation is also a crucial point. For example, in the case of Srs2, how do regulatory factors restrain Srs2 from dismantling all Rad51 filaments thus preventing recombination from taking place altogether? Emerging studies of Srs2 implicate Rad52, Rad55-Rad57, and the SHU complex as negative regulators of Srs2 antirecombinase activity, but we still lack a complete picture of how these factors work together to allow for a physiologically sensible outcome. Interestingly, in vivo and in vitro studies have found that the meiosis-specific recombinase Dmc1 completely blocks the ability of Srs2 to translocate on ssDNA, thus rendering Dmc1 fully resistant to its antirecombinase activity [144]. However, there is no clear biological explanation for why Srs2 might be inhibited by Dmc1, or even how Dmc1 inhibits Srs2 whereas Rad51 cannot. Similar types of regulatory questions can be posed for many different helicases.

Fully understanding helicase regulation will also require more detailed investigations into the full spectrum of potential protein-protein interactions. For example, in a two-hybrid analysis of Srs2, more than 70 proteins including major HR-related factors, such as the Mre11 nuclease and the Sgs1 and Mph1 helicases, were identified as Srs2 interactors [145]. It is not yet clear why Srs2 interacts with such a large number of proteins. Curiously, there does not appear to be a close homolog of Srs2 in mammalian cells, although FBH1 and PARI are possible candidates [78,79,80,146]. In addition, there are other human helicases (e.g., RECQ5, BLM) capable of removing RAD51 from DNA [6]. So, it is possible that the antirecombinase activity of Srs2 has been co-opted by one of these other unrelated helicases. Thus, an important problem moving forward will be to more clearly define how these human enzymes participate in recombination and how they are regulated.

A complete mechanistic understanding of Pif1 in DNA synthesis during BIR is also an important future goal. For example: What dictates the speed of BIR and why is it so much slower than normal DNA replication? Given that Pif1 is the major helicase that participates in DNA synthesis during BIR, one possibility is that the translocation velocity of Pif1 may be rate limiting for DNA replication. In addition, replication forks associated with BIR appear to be highly susceptible to conflicts with the transcription machinery [137]. Thus, it would be important to know if this limitation is due to some physical property of Pif1 in comparison to the normal S-phase MCM replicative helicase. Finally, it will be important to more fully define the organization of Pif1 in the BIR replication fork. For example, it will be important to define how many molecules of Pif1 are necessary and establish where they are located with the BIR migrating bubble. We have focused on Pif1′s roles in the nucleus, but it is important to remember that the most prominent *pif1Δ* phenotype is a mitochondrial defect [49]. Although the mechanism by which Pif1 participates in the maintenance of mitochondrial DNA (mtDNA) is poorly understood, it has been proposed that Pif1 recognizes and resolves particular DNA structures in A/T-rich sections of mitochondrial DNA [147]. Thus, an additional question centers on more fully defining the role of Pif1 in mtDNA maintenance.

## 6. Concluding Remarks

In this review we have discussed the mechanisms and biological functions of Sf1a and Sf1b helicases. A central theme is that despite the diversity in functions, structures, and mechanisms, the Sf1a and Sf1b helicase subfamilies share many interesting mechanistic and structural features. While we have placed our emphasis on the *S. cerevisiae* proteins Srs2 and Pif1, mutations in both Sf1a and Sf1b helicases in humans can lead to genetic disorders, and mutations in the yeast proteins may reflect these disorders. Therefore, future studies of these yeast proteins, as well as other types of helicases, offer the potential for deep insights into the molecular defects underlying human diseases.
